# Stacked PZT Discs Generate Necessary Power for Bone Healing through Electrical Stimulation in a Composite Spinal Fusion Implant

**DOI:** 10.3390/bioengineering5040090

**Published:** 2018-10-23

**Authors:** Eileen S. Cadel, Ember D. Krech, Paul M. Arnold, Elizabeth A. Friis

**Affiliations:** 1 Bioengineering Graduate Program, University of Kansas, Lawrence, KS 66045, USA; ecadel607@gmail.com (E.S.C.); ekrech@ku.edu (E.D.K.); 2 Department of Neurosurgery, University of Kansas Medical Center, Kansas City, MO 66160, USA; parnold@kumc.edu; 3 Department of Mechanical Engineering, University of Kansas, Lawrence, KS 66045, USA

**Keywords:** electrical stimulation, piezoelectric composites, bone healing, power generation, human powered implants, novel spinal interbody implants

## Abstract

Electrical stimulation devices can be used as adjunct therapy to lumbar spinal fusion to promote bone healing, but their adoption has been hindered by the large battery packs necessary to provide power. Piezoelectric composite materials within a spinal interbody cage to produce power in response to physiological lumbar loads have recently been investigated. A piezoelectric macro-fiber composite spinal interbody generated sufficient power to stimulate bone growth in a pilot ovine study, despite fabrication challenges. The objective of the present study was to electromechanically evaluate three new piezoelectric disc composites, 15-disc insert, seven-disc insert, and seven-disc Compliant Layer Adaptive Composite Stack (CLACS) insert, within a spinal interbody, and validate their use for electrical stimulation and promoting bone growth. All implants were electromechanically assessed under cyclic loads of 1000 N at 2 Hz, representing physiological lumbar loading. All three configurations produced at least as much power as the piezoelectric macro-fiber composites, validating the use of piezoelectric discs for this application. Future work is needed to characterize the electromechanical performance of commercially manufactured piezoelectric stacks under physiological lumbar loads, and mechanically assess the composite implants according to FDA guidelines for lumbar interbody fusion devices.

## 1. Introduction

Upwards of 30% of the general population will experience low back pain due to degenerative disc disease, with even higher incidence rates for those over the age of 45 [1]. For those whose pain cannot be controlled using conservative treatment options, lumbar spinal fusion is the most common surgical treatment used to alleviate pain associated with severe disc degeneration [2]. In 2016, over 417,000 thoracolumbar fusion procedures were performed in the U.S., with success rates as high as 90% [3,4]. However, difficult-to-fuse patients (i.e., smokers and diabetics) make up more than half of spinal fusion patients and experience significantly lower success rates due primarily to pseudarthrosis [5,6,7,8].

To address the lower success rates in the difficult-to-fuse patient population, adjunct therapies have been used to supplement bone growth and increase bone healing. Bone morphogenetic protein (BMP) can be combined with bone grafts to help recruit osteoblasts and encourage differentiation to promote bone formation, but have been associated with adverse events [9,10]. Ultrasound has been used for several decades as an adjunct therapy to spinal fusion to promote bone formation, but it relies on patient adherence to the prescribed therapy [11,12]. More recently, the use of adipose-derived stem cells have been investigated to promote bone regeneration; however, there are still many obstacles to overcome before it can be used clinically [13].

In 1957, Fukada and Yasuda discovered that bone exhibits piezoelectric properties; electric charge is generated as a result of applied mechanical stress [14]. In 1962, Bassett and Becker continued to build on this concept and found that compressive loading of bone generates a negative charge that stimulates the bone remodeling process [15]. Because lumbar spinal fusions typically fail due to pseudarthrosis, negative direct current (DC) electrical stimulation was investigated as an adjunct therapy to promote additional bone formation and eliminate motion across the joint [16]. The most common internal electrical stimulation device for spinal fusion applications delivers current to implanted titanium electrodes, which are placed bilaterally along the transverse processes after bone is placed in this space. Current delivered to these electrodes is generated by an implantable battery pack [17]. This adjunct therapy emerged in the 1980s and has been shown to be clinically safe and effective, with generally positive outcomes. Several drawbacks have hindered its adoption: increased cost, patient discomfort, and infection. Complications arising from this device necessitate its removal, which requires a second surgery [18,19,20,21,22,23,24].

Previous studies have investigated using a piezoelectric macro-fiber composite, as the material for spinal interbody implants, to supply the necessary power to deliver electrical stimulation to the site of lumbar fusion [25,26,27]. These composites allowed the piezoelectric (PZT) macro fibers to strain in response to typical lumbar spinal loads and produce an electrical charge, and the matrix material added the needed toughness that the brittle PZT lacks. Goetzinger et al. found that increasing the number of layers of PZT elements, connected electrically in parallel and stacked mechanically in series, decreased source impedance of the implant and produced sufficient power needed for 4–5 µA cm^2^ of current density [25]. Friis et al. reported the preclinical success of these PZT macro-fiber composite implants to stimulate bone growth and increase spinal fusion rates in a four-month pilot ovine study [27]. However, the fabrication method used limited the layer thickness of the composite to 1 mm and was difficult to replicate and scale-up.

PZT discs show promise as a material to generate power within an interbody implant due to their compressive strength and versatility. Layers of thin PZT discs, which are connected electrically in parallel and stacked mechanically in series, have been shown to produce 1–2 mW power at human walking loads and frequencies [28,29,30,31]. Recently, incorporating PZT discs in a composite for low-frequency power generation has been investigated, and the addition of a compliant layer between PZT discs, a CLACS (Compliant Layer Adaptive Composite Stack) structure, was shown to significantly increase power generation [32]. However, PZT disc composites have never been used as a generator for internal electronegative stimulation with the goal of increasing bone formation. Therefore, power from physiological loading of these composites must be characterized and evaluated using the using the power generated from the PZT macro-fiber composites as a success threshold.

The aim of this study was to evaluate electromechanical properties of three composite PZT disc stacks as inserts within a spinal interbody implant, and validate their use as a generator for electrical stimulation: (1) 15-disc implant; (2) seven-disc implant; (3) seven-disc CLACS implant. It is hypothesized that increasing total PZT volume will result in increased power, the seven-disc CLACS implant will produce more power than the seven-disc implant, and that the 15-disc implant will produce at least as much power as PZT macro-fiber composites for a similar PZT volume and ratio of surface area of PZT to implant footprint surface area.

## 2. Materials and Methods

### 2.1. Implant Design

**Figure 1:**
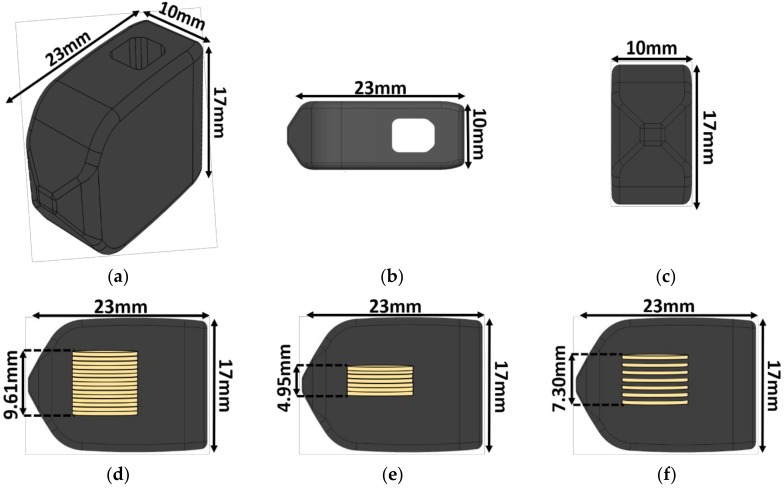
Implant schematic. The 23 × 10 × 17 mm transforaminal lumbar interbody fusion (TLIF) implant: (**a**) Isometric view, (**b**) top view, (**c**) front view, (**d**) side view with the 15-disc insert, (**e**) side view with the seven-disc insert, and (**f**) side view with the seven-disc CLACS (Compliant Layer Adaptive Composite Stack) insert. The outer shape remained constant regardless of the insert type.

The implant size and shape chosen for this study was modeled after a transforaminal lumbar interbody fusion (TLIF) implant previously cleared by the U.S. Food and Drug Administration (FDA), with dimensions of 23 × 10 × 17 mm and a graft window size of 5 × 5.5 × 17 mm (Figure 1). This configuration represents the aspect ratio, of height to footprint, that corresponds to the mechanical worst-case typically used in mechanical testing for FDA clearance. The total volume of the implant was calculated to be 3013.95 mm^3^ using the SOLIDWORKS mass properties tool. A mold of the implant was made using a to-scale 3D printed replica and high-performance liquid silicone (Dragon Skin 10, Smooth-On, Macungie, PA, USA).

### 2.2. Piezoelectric Composite Material Fabrication

Three different piezoelectric inserts (*n* = 6 for each insert type) were made using pre-poled and electroded 7 × 0.4 mm discs of modified PZT-4 (Lead Zirconate Titanate, SMD7T04R111, STEMiNC, Doral, FL, USA). The diameter of the discs was chosen to fit within the front end of the implant while maximizing the ratio of PZT cross-sectional surface area to implant footprint surface area. The number of PZT discs was chosen so that the three different inserts could fit in all TLIF implant configurations, with heights ranging from 11 mm to 17 mm.

**Figure 2:**
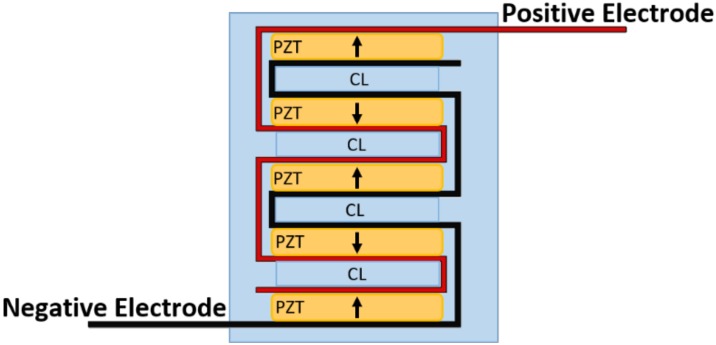
CLACS schematic. Piezoelectric (PZT) discs were connected electrically in parallel using two strips of copper foil, acting as the positive and negative electrodes, and silver conductive epoxy. The discs were then stacked mechanically in series with 0.4 mm compliant layers (CL) of matrix epoxy between each PZT disc.

The first configuration was a 15-disc insert—15 PZT discs connected electrically in parallel and stacked mechanically in series. The second configuration was a seven-disc CLACS insert—seven PZT discs connected electrically in parallel and stacked mechanically in series, with a 0.4 mm compliant layer of matrix epoxy between each disc (Figure 2). The 0.4 ± 0.02 mm compliant layers of matrix epoxy were cut using a precision section saw. The final configuration was a seven-disc insert—seven discs connected electrically in parallel and stacked mechanically in series. The method for stacking discs was adapted from Krech et al. [32]. All discs were connected electrically in parallel using two strips of copper foil 0.02 mm in thickness (Basic Copper, Carbondale, IL, USA) and silver conductive epoxy (EPO-TEK H20E, Epoxy Technology, Billerica, MA, USA), then cured for 2 h at 100 °C. The discs were then stacked mechanically, in series, using a medical grade matrix epoxy and inspected to ensure mechanical bond was created between all layers (EPO-TEK 301, Epoxy Technology, Billerica, MA, USA). The matrix epoxy was cured at room temperature for 24 h, followed by a 2 h cure at 65 °C to ensure crosslinking of the material.

Once fully cured, each insert was placed inside the silicone mold (Dragon Skin 10 Medium, Smooth-On, Macungie, PA, USA) on top of a 7 × 7 × 4 mm slice of cured matrix epoxy to ensure a uniform distance from the bottom of the implant for all samples. The end of each copper strip was fed through a small horizontal slit at the front end of the implant in the silicone molds. The molds were then filled with the medical grade matrix epoxy (EPO-TEK 301, Epoxy Technology, Billerica, MA, USA). This epoxy was chosen to encapsulate the implants due to its similarity in storage modulus to PEEK (polyether-ether-ketone), and because it has previously been used in physiological load-bearing applications [25,33,34,35]. The implants were cured at room temperature for 24 h, then removed from their molds and oven cured at 65 °C for 2 h.

### 2.3. Electromechanical Testing

To simulate physiological loading conditions in the lumbar spine, each implant was subjected to a pure compressive 1200 N preload, followed by peak-to-peak cyclic load of 1000 N at 2 Hz for 15 cycles using an MTS MiniBionix 858 (MTS, Eden Prairie, MN, USA) with a sampling frequency of 512 Hz. The 2 Hz frequency was chosen to best represent normal human gait [36]. The voltage was measured across 31 different applied resistances in series, with the implant ranging from 16.0 kΩ to 63.4 MΩ, to fully characterize the voltage and power capabilities of the implants. The loading and frequency profiles were chosen to represent loads experienced between vertebral bodies in the lumbar spine with posterior instrumentation from normal human walking [30,37,38].

### 2.4. Data Analysis

**Figure 3:**
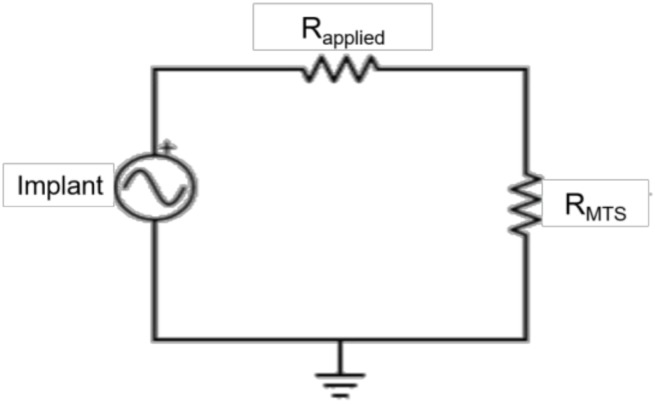
Circuit diagram. The implants were connected in series with the applied resistances (*R_applied_*) and in parallel with the MTS. A voltage divider was used to calculate the voltage produced by the implant, scaled by the applied resistance and the resistance of the MTS (*R_MTS_*).

Customized MATLAB software (Mathworks, Natick, MA, USA) was used to calculate power produced by each implant from the measured voltages. The average maximum voltage was calculated using a voltage divider (Figure 3) from the amplitude of the five middle loading cycles, and scaled by the corresponding applied resistive load (*R_applied_*) and the 2 MΩ resistance of the MTS (*R_MTS_*) (Equation (1)) [39].

(1) $$\;V_{scaled}=V_{MTS}*\frac{1+R_{applied}}{R_{MTS}}\;$$

To calculate the power produced by each implant, the voltage was converted to RMS then power was calculated using Joule’s Law (Equation (2)) [28,39].

(2) $$\;P=\frac{V_{RMS}^{2}}{R_{total}}\;$$

To calculate power density, the power was divided by the total volume of PZT material for each implant. A two-way ANOVA with a Tukey–Kramer post-hoc analysis was used to determine statistical significance between power and power density produced by the three implant types at each applied resistance (α = 0.05). A one-way ANOVA with a Tukey–Kramer post-hoc analysis was used to determine the effect of implant type at 30 MΩ, which was the resistance of the rectifying circuit used in the pilot ovine study to transform the signal from AC to DC (α = 0.05).

## 3. Results

**Figure 4:**
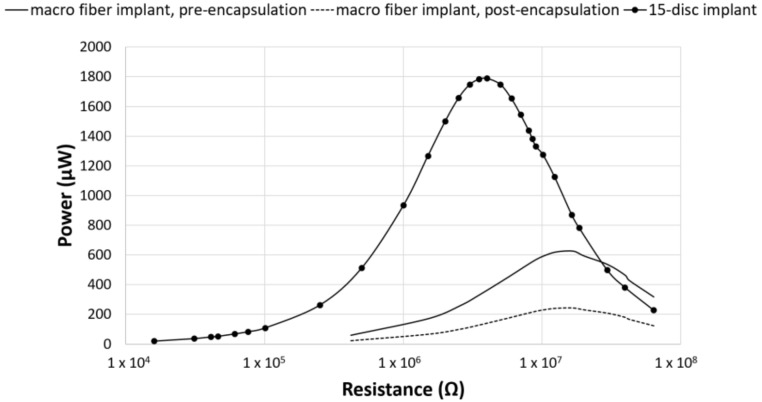
Power comparison between PZT fibers and PZT discs. Power generated by the composite macro-fiber implants from Goetzinger et al. (pre-encapsulated implant) and Tobaben et al. (post-encapsulated implant) compared to the TLIF implant with a 15-disc insert [25,26]. For similar PZT volumes and cross-sectional area ratios, the composite 15-disc implant outperformed the macro-fiber implants.

In contrast to previous studies, investigating piezoelectric composite spinal fusion implants that used a nine-layer stack of 48 PZT macro fibers, the current study investigated the impact on power output when switching to a stack of PZT discs [25]. To validate this switch, the average power output from the 15-disc implants was compared to previous studies that assessed power production from a composite TLIF throughout the manufacturing process [25,26]. The total volume of PZT in the macro fiber implants was 217 mm^3^. This was very similar to the 230 mm^3^ volume of PZT in the 15-disc implants. Across the resistance sweep, the 15-disc implant outperformed both the pre-encapsulated and post-encapsulated TLIF implants (Figure 4) [25,26]. The ratio of PZT cross-sectional area to footprint surface area, for the pre-encapsulation and post-encapsulated macro-fiber implant, was 30% and 16%, respectively, as compared to 27% for the disc implants. The PZT disc implants generated more power, compared to the macro-fiber implant, for similar PZT volumes and cross-sectional area to footprint ratios. This was due to the increased number of layers connected electrically in parallel, which lowered the source impedance of the insert, and the improved materials and fabrication methods.

**Figure 5:**
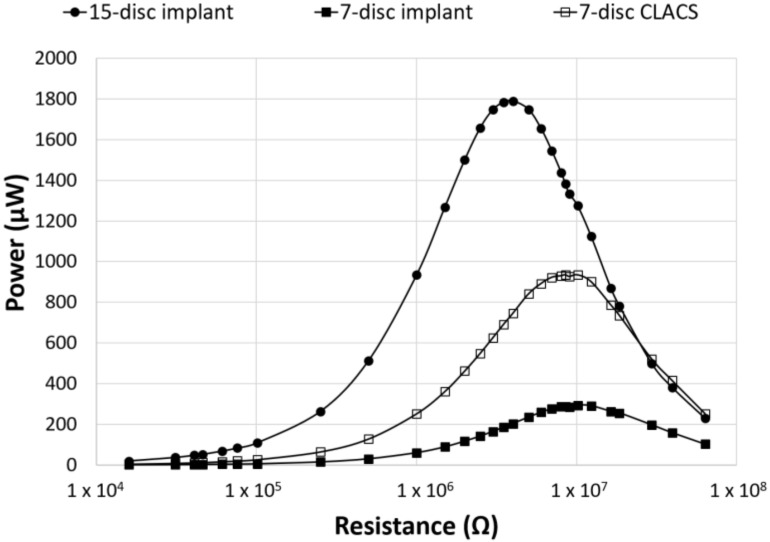
Power as a function of applied resistance and PZT disc implant type. Power generated for the three implant configurations across an applied resistive load from 16.0 kΩ to 63.4 MΩ. The seven-disc CLACS implant produced more power than the seven-disc implant for the same volume of PZT. The effect of the PZT volume and compliant layer on the power generated was clearly illustrated.

The seven-disc CLACS were manufactured to address potential deficits in mechanical properties due to the brittle nature of the 15-disc implants. The power for the seven-disc CLACS implants was compared to both the 15-disc implants and the seven-disc implants. The seven-disc implants and seven-disc CLACS implants had identical PZT volume and surface area ratios (Figure 5). As expected, the power increased as the applied resistive load increased until the applied resistive load matched the impedance of each implant, demonstrating maximum power output for each implant. Table 1 summarizes the voltage and applied resistive load corresponding to maximum power produced by each implant configuration. A two-way ANOVA was used to determine the effect of implant type and applied resistance on power. The log transform of power was used to satisfy normality and variance requirements for this analysis. The interaction between implant type and applied resistance was significant (*p* < 0.01), indicating that the effect of resistance on the power generated depended on the type of implant. As resistance increased, the effect of insert type decreased (*p* > 0.05).

**Figure 6:**
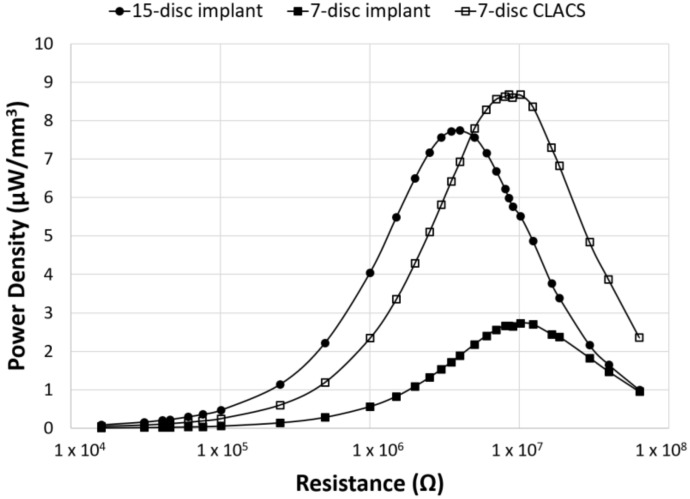
Power density as a function of applied resistance and PZT disc implant type. Power density (power normalized by volume of PZT for each implant type) generated for the three implant configurations across an applied resistive load, from 16.0 kΩ to 63.4 MΩ. The CLACS structure allowed for more power to be produced per unit volume of PZT.

**Figure 7:**
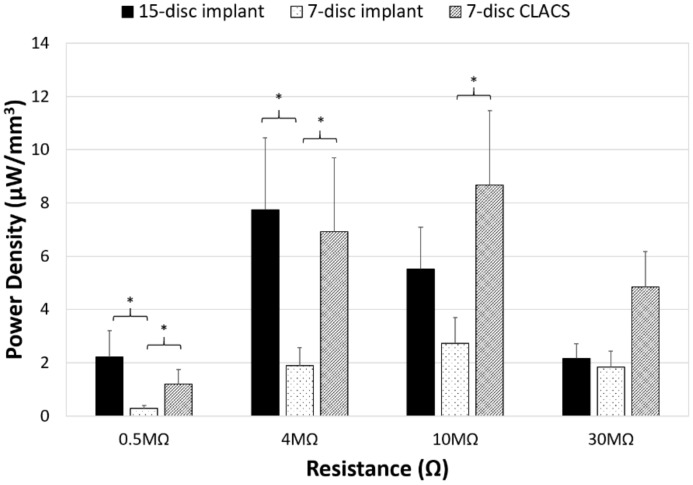
Power density comparison between PZT disc implants. Power density (power normalized by volume of PZT for each implant type) generated for the three implant configurations (15-disc implant, seven-disc implant, and seven-disc CLACS implant) at a low applied resistive load (0.5 MΩ), resistance of maximum power for the 15-disc implant (4 MΩ), resistance of maximum power for both seven-disc implants (10 MΩ), and at a high applied resistive load (30 MΩ). * Represents significant difference (*p* < 0.05). The largest effect of implant type was observed closest to the resistance corresponding to maximum power.

To compare power across implants with different insert types, the power was normalized by the total PZT volume to obtain power density for each implant configuration (Figure 6). Though maximum power occurred at the lowest resistive load for the 15-disc implant, the maximum power density was greatest for the seven-disc CLACS implant. The 15-disc implant had the largest power density at resistances lower than 4 MΩ, the resistance corresponding to its maximum power. Figure 7 illustrates the power density of each implant at 0.5 MΩ, 4 MΩ, 10 MΩ, and 30 MΩ corresponding to a low applied resistance, resistance at maximum power for the 15-disc implant, resistance at maximum power for both seven-disc implant types, and a high applied resistive load, respectively. As resistance increased, the effect of implant type on power density decreased, following the same statistical trend as the power results.

## 4. Discussion

The purpose of this study was to assess the power producing capabilities of PZT stacked disc inserts in a TLIF implant cage under physiological loads present in the lumbar spine and determine if they are sufficient to produce the necessary power needed to stimulate bone growth. The use of PZT stacks as a generator of power at frequencies of human body motion is a newly emerging field. Previous work has evaluated the use of PZT composites to power in vivo devices, such as embedded MEMS devices and wearable sensors [28,29,40]. Using the generated current of such devices to produce electrical stimulation to increase bone healing in lumbar spinal fusion has only recently been studied [25,26,27,41]. These studies utilized PZT macro fibers embedded in an epoxy matrix to improve the mechanical properties of the brittle ceramic. However, these composites had poor interface strength between the fibers and epoxy, as well as fabrication and supply chain difficulties. Despite these limitations, this composite structure did produce sufficient power to stimulate bone growth [27]. The goal of switching to the PZT disc structure was to address several of the fabrication and power production efficiency limitations, found with macro fibers, while generating at least as much power as the macro-fiber composites.

One consideration when switching from the PZT macro fibers to the PZT discs was to ensure that the PZT disc inserts would fit within a TLIF interbody implant. Accordingly, the TLIF interbody implant design used in this study had a defined length and width of 17 × 10 mm but a variable height of 11 to 17 mm. To streamline future manufacturing of all implant size iterations, one defined PZT insert size will likely be chosen to fit in the entire height range of interbody implants required. The maximum diameter of the PZT disc was limited to 7 mm and the maximum height of the PZT insert was 10 mm to ensure even encapsulation above and below the insert. Given these constraints and the desire to maximize the amount of PZT, 15 (7 × 0.4 mm) PZT discs were used in the first implant configuration, the 15-disc implants. Seven (7 × 0.4 mm) PZT discs with a 0.4 mm layer of epoxy between each disc, the seven-disc CLACS implants, were chosen for the second implant configuration because of its similar overall height to the 15-disc insert and the enhanced power production [32]. To compare the power output for the same volume of PZT to the seven-disc CLACS implants, the final insert configuration had seven (7 × 0.4 mm) PZT discs.

As seen in Figure 4, the 15-disc implant was able to produce substantially more power across the resistance sweep as compared to the pre- and post-encapsulated PZT macro-fiber implants, for similar PZT volumes, despite a 5% decrease in PZT surface area to implant footprint [25,26]. This increase in power could be attributed to either the PZT discs themselves or the fabrication method used to produce the PZT disc composite implants. The PZT disc composite implants were much more efficient at converting cyclic loads to power. Additionally, the resistance corresponding to maximum power was lower for the 15-disc implant and it did not exhibit the same lack of interface strength, fabrication, or PZT supply chain problems as the macro-fiber implants.

A previous study investigated the effect of adding a complaint layer between layers of PZT discs to increase power production [32]. Power generation was compared between the 15-disc implant, seven-disc implant, and seven-disc CLACS implant. As expected, the addition of the compliant layer did not change the resistance corresponding to maximum power of the implant, but it did increase the maximum power produced for the same volume of PZT. The large standard deviations for power and voltage can most likely be attributed to the rudimentary method used to produce the prototype PZT disc stacks. Commercially manufactured stacks of PZT discs that are co-fired to an electrode could reduce this variability. However, the addition of compliant layers between the discs would not be possible with current commercial manufacturing methods. The use of a co-fired stack and its power generation potential for in vivo use, and other more robust CLACS fabrication methods, should be further investigated.

The two-way ANOVA for the log of power for the 15-disc, seven-disc, and seven-disc CLACS implants resulted in a significant interaction between implant type and applied resistive load (*p* < 0.01). This implies that the amount of power generated by each implant type is highly dependent on the applied resistive load and that this effect is largest at resistances below insert impedance, the resistance corresponding to maximum power. The current produced by the piezoelectric implant will need to be rectified to an electronegative signal. This will require a signal conditioning circuit incorporated into the TLIF implant. Smaller electrical components have lower resistances. Therefore, the resistance corresponding to this circuit will most likely take advantage of the compliant layer effect.

For the same PZT volume and ratio of PZT surface area to implant footprint, the seven-disc CLACS implant produced significantly more power than the seven-disc implant for every resistance (*p* < 0.05). At 10 MΩ, the resistance corresponding to maximum power, there was a 217% increase in power between the seven-disc and the seven-disc CLACS implants (*p* = 0.01). It is hypothesized that the interdigitated compliant layers resulted in increased strain on each layer of PZT, resulting in more power under the same loading conditions [30]. However, the exact effect of the compliant layer is difficult to predict because of the complex strain generated in the PZT due to the presence of the compliant layers [32]. Quantifying the increased strain and corresponding power would be beneficial to inform future implant design decisions. Future work should further investigate the effect of compliant layers on mechanical and material properties.

The two-way ANOVA on the log of power density for the implants resulted in similar power densities (*p* > 0.05) across the resistance sweep between the 15-disc and the seven-disc CLACS implants, despite the 15-disc implant having more than twice the volume of PZT. This further supports the notion that the compliant layer improves electromechanical coupling per unit volume of PZT material. Additionally, for resistances greater than the impedance of the 15-disc implant, the seven-disc CLACS implant produced more power per unit volume of PZT (Figure 6). Similar to the previous results, the two-way ANOVA resulted in a significant interaction between implant type and applied resistive load (*p* < 0.01). This is likely attributed to the mismatch in insert resistance corresponding to maximum power generated between the implant configurations. Though the seven-disc CLACS implants had the greatest overall power density, the maximum power occurred at 10 MΩ as compared to the 15-disc implants that produced maximum power at 4 MΩ. This offset in power exacerbated the difference in power density between these implants.

The impact of this study is that PZT discs can generate sufficient power needed to stimulate bone healing. However, many design choices are necessary to develop a TLIF implant with a PZT disc insert. In previous work, a circuit with a resistance of 30 MΩ was used to rectify the power produced by the piezoelectric material to a DC signal. For the three implant types investigated in this study, the power and voltage produced at this resistance level can be seen in Table 2. The 15-disc implant and seven-disc CLACS implant produced significantly more power than the seven-disc implant (*p* < 0.05). The power produced by the 15-disc implant and seven-disc CLACS implant were statistically similar. Although the 15-disc implant and seven-disc CLACS implant produced the most power, they also produced the most voltage, which could add complexity to the implant circuity. While the voltage measurements were not the focus of this study, voltage does have a large effect on the electrical components that are necessary to transform the AC output, associated with human motion, to an electronegative DC output, which is necessary to stimulate bone growth. Understanding the effect of PZT implant design choices on both the power and voltage produced by implants with varying circuit components will allow for a more informed design of TLIF implants that are capable of producing DC stimulation.

These findings have significant clinical relevance. Lumbar fusion rates remain frustratingly low, and current surgical management has not been able to achieve acceptable outcomes. Recombinant human protein, Rh-BMP-2, currently used off-label in a TLIF interbody, can be used as adjunct therapy to spinal fusion to improve fusion rates. However, it adds significant cost and is associated with several complications, including ectopic bone growth and radiculitis. Other synthetics and bone graft substitutes have not demonstrated high fusion rates. A piezoelectric composite spinal fusion cage offers a potential solution to low fusion rates. It can be placed with standard surgical techniques, has a low risk profile, and will likely be well tolerated by patients. 

## 5. Conclusions

Three different configurations of PZT disc inserts, limited by the size of a 23 × 10 × 17 mm TLIF implant, successfully produced the sufficient power needed to stimulate bone growth, using the power generated by the macro-fiber implants as the success threshold. Incorporating this technology into a TLIF would be advantageous for patients undergoing lumbar fusion, particularly for the difficult-to-fuse patient population who would benefit from improved healing. As expected, greater PZT volume resulted in significantly greater power generation. The use of compliant layers between the PZT discs also enhanced power generation. Within a TLIF implant design, PZT discs successfully produced more power than PZT macro fibers for similar ratios of PZT surface area to implant footprint and similar volumes of PZT. Future work should further characterize the power and voltage of co-fired PZT stacks and mechanically assess PZT implants according to FDA recommended guidelines for lumbar interbody fusion devices.

## Figures and Tables

**Table 1:** Average maximum power and voltage ± standard deviation.

Implant Type	Average Maximum Power (μW)	Average Voltage at Maximum Power (V)	Applied Resistive Load for Maximum Power (MΩ)
15-disc insert	1789 ± 540	84 ± 12	4
seven-disc insert	294 ± 90	54 ± 9	10
seven-disc CLACS insert	935 ± 261	96 ± 14	10

**Table 2:** Power and voltage for a given implant rectifying circuit with a resistance of 30 MΩ. * Represents significant difference from the seven-disc insert (*p* < 0.05).

Implant Type	Average Power at Circuitry Resistance (μW)	Average Voltage at Circuitry Resistance (V)
15-disc insert	500 ± 108 *	121 ± 13 *
seven-disc insert	197 ± 56	75 ± 11
seven-disc CLACS insert	521 ± 125 *	123 ± 15 *
